# Dark control: The default mode network as a reinforcement learning agent

**DOI:** 10.1002/hbm.25019

**Published:** 2020-06-05

**Authors:** Elvis Dohmatob, Guillaume Dumas, Danilo Bzdok

**Affiliations:** ^1^ Criteo AI Lab Paris France; ^2^ INRIA, Parietal Team Saclay France; ^3^ Neurospin, CEA Gif‐sur‐Yvette France; ^4^ Institut Pasteur, Human Genetics and Cognitive Functions Unit Paris France; ^5^ CNRS UMR 3571 Genes, Synapses and Cognition, Institut Pasteur Paris France; ^6^ University Paris Diderot, Sorbonne Paris Cité Paris France; ^7^ Centre de Bioinformatique, Biostatistique et Biologie Intégrative Paris France; ^8^ Department of Biomedical Engineering, McConnell Brain Imaging Centre, Montreal Neurological Institute, Faculty of Medicine, School of Computer Science McGill University Montreal Canada; ^9^ Mila—Quebec Artificial Intelligence Institute Montreal Canada

**Keywords:** artificial intelligence, human intelligence, systems neuroscience

## Abstract

The default mode network (DMN) is believed to subserve the baseline mental activity in humans. Its higher energy consumption compared to other brain networks and its intimate coupling with conscious awareness are both pointing to an unknown overarching function. Many research streams speak in favor of an evolutionarily adaptive role in envisioning experience to anticipate the future. In the present work, we propose a *process model* that tries to explain *how* the DMN may implement continuous evaluation and prediction of the environment to guide behavior. The main purpose of DMN activity, we argue, may be described by Markov decision processes that optimize action policies via value estimates through vicarious trial and error. Our formal perspective on DMN function naturally accommodates as special cases previous interpretations based on (a) predictive coding, (b) semantic associations, and (c) a sentinel role. Moreover, this process model for the neural optimization of complex behavior in the DMN offers parsimonious explanations for recent experimental findings in animals and humans.

## 1 INTRODUCTION

In the absence of external stimulation, the human brain is not at rest. At the turn to the 21st century, brain‐imaging may have been the first technique to allow for the discovery of a unique brain network that would subserve baseline mental activities (Buckner, Andrews‐Hanna, & Schacter, 2008; Bzdok & Eickhoff, 2015; Raichle et al., 2001). The “default mode network” (DMN) continues to metabolize large quantities of oxygen and glucose energy to maintain neuronal computation during free‐ranging thought (Fiser, Chiu, & Weliky, 2004; Kenet, Bibitchkov, Tsodyks, Grinvald, & Arieli, 2003). The baseline energy demand is only weakly modulated at the onset of defined psychological tasks (Gusnard & Raichle, 2001). At its opposite, during sleep, the decoupling of brain structures discarded the idea of the DMN being only a passive network resonance and rather supported an important role in sustaining conscious awareness (Horovitz et al., 2009).

This *dark matter of brain physiology* (Raichle, 2006) begs the question of the biological purpose underlying neural activity in the DMN. Its time dynamics, however, still remain elusive at the electrophysiological level (Baker et al., 2014; Brookes et al., 2011; De Pasquale et al., 2010). What has early been described as the “stream of consciousness” in psychology (James, 1890) found a potential neurobiological manifestation in the DMN (Raichle et al., 2001; Shulman et al., 1997). Axonal tracing injection in such parts of the association cortex in monkeys were shown to resemble connectivity links between nodes of the human DMN (see here for details on anatomical connections: [Buckner et al., 2008]). Additionally, myelination patterns of axon connections were found to complete particularly late in these cortical areas (Flechsig, 1920), often believed to reflect sophistication of subserved neural processes (Sowell et al., 2003; Yakovlev, 1967). We propose that this set of some of the most advanced regions in the association cortex (Margulies et al., 2016; Mesulam, 1998) are responsible for higher‐order control of human behavior (Bzdok et al., 2015). Our perspective therefore follows the notion of “a hierarchy of brain systems with the DMN at the top and the salience and dorsal attention systems at intermediate levels, above thalamic and unimodal sensory cortex” (Carhart‐Harris & Friston, 2010).

## 2 TOWARD A FORMAL ACCOUNT OF DEFAULT MODE FUNCTION: HIGHER‐ORDER CONTROL OF THE ORGANISM

The network nodes that compose the human DMN are hubs of high baseline neural activity. These regions typically decrease when engaged in well‐defined psychological experiments (Gusnard & Raichle, 2001). The standard mode of neural information maintenance and manipulation has been argued to mediate evolutionarily conserved functions (Binder et al., 1999; Brown, 1914; Buzsáki, 2006). Today, many psychologists and neuroscientists believe that the DMN implements some form of probabilistic estimation of past, hypothetical, and future events (Binder, Desai, Graves, & Conant, 2009; Buckner et al., 2008; Fox et al., 2005; Hassabis, Kumaran, Vann, & Maguire, 2007; Schacter, Addis, & Buckner, 2007; Spreng, Mar, & Kim, 2009), even if spatially overlapping neural activity responses do not imply identical neuronal computations (Kernbach et al., 2018; Wang et al., 2018; Woo et al., 2014). This brain network might have emerged to continuously predict the environment using mental imagery as an evolutionary advantage (Suddendorf & Corballis, 2007). However, information processing in the DMN has also repeatedly been shown to directly impact human behavior. Goal‐directed task performance improved with decreased activity in default mode regions (Weissman, Roberts, Visscher, & Woldorff, 2006) and increased DMN activity was linked to more task‐independent, yet sometimes useful thoughts (Mason et al., 2007; Seli, Risko, Smilek, & Schacter, 2016). Gaining insight into DMN function is particularly challenging because this brain network appears to simultaneously influence perception‐action cycles in the present and to support mental travel across time, space, and content domains (Boyer, 2008).

We aim at proposing an alternative to reasoning about the DMN based on longstanding cognitive theory. The present work adopts the control‐theoretical perspective of a human *agent* faced with the choice of the next actions guided by outcomes to optimize behavioral performance. These outcomes can be really experienced, hypothetically imagined, or expected in the future. Formally, we propose reinforcement learning (RL) as a particularly attractive framework for describing, containing, and quantifying the unknown function underlying DMN activity. An intelligent agent improves the interaction with the environment by continuously updating its computation of value estimates and action predispositions through integration of feedback outcomes. That is, “[agents], with their actions, modify the environment and in doing so partially determine their next stimuli, in particular stimuli that are necessary for triggering the next action” (Pezzulo, 2011). Agents with other behavioral policies therefore sample different distributions of action‐perception trajectories (Ghavamzadeh, Mannor, Pineau, Tamar, et al., 2015). Henceforth, *control* refers to the influence that an agent exerts by interacting with the environment to reach preferred states.

At the psychological level, the more the ongoing executed task is unknown and unpracticed, the less stimulus‐independent thoughts occur (Christoff, Irving, Fox, Spreng, & Andrews‐Hanna, 2016; Filler & Giambra, 1973; Teasdale et al., 1995). Conversely, it has been empirically shown that, the more the world is easy to foresee, the more human mental activity becomes detached from the actual sensory environment (Antrobus, Singer, & Greenberg, 1966; Mason et al., 2007; Pope & Singer, 1978; Weissman et al., 2006). Without requiring explicit awareness, these “offline” processes may contribute to optimizing control of the organism in general. We formalize a *policy matrix* to capture the space of possible actions that the agent can perform on the environment given the current state. A *value function* maps environmental objects and events (i.e., states) to expected reward outcomes. Switching between states reduces to a sequential processing model. Informed by outcomes of performed actions, neural computation reflected in DMN dynamics could be constantly shaped by prediction error through feedback loops. The present computational account of DMN function will be described in the mathematical framework of Markov decision processes (MDP). MDPs specifically formalize decision making in stochastic contexts with reward feedback, which becomes available intermittently.

Such a RL perspective on DMN activity can naturally embed human behavior into the tension between exploitative action with immediate gains and exploratory action with longer‐term gratification. We argue that DMN implication in many of the most advanced human capacities can be recast as prediction error minimization informed by internally generated probabilistic simulations—“covert forms of action and perception” (Pezzulo, 2011)—, allowing maximization of action outcomes across different time scales. Such a purposeful optimization objective may be solved by a stochastic approximation based on a brain implementation of Monte Carlo sampling. Even necessarily imperfect memory recall, random day‐time mind‐wandering, and seemingly arbitrary dreams during sleep may provide randomly sampled blocks of pseudo‐experience that are instrumental to iteratively optimize the behavioral agenda of the organism.

Evidence from computational modeling of human behavior (Kording & Wolpert, 2004) and cell recording experiments in ferrets (Fiser et al., 2004) suggest that much of brain activity is dedicated to “the development and maintenance of [a] probabilistic model of anticipated events” (Raichle & Gusnard, 2005). The present article proposes a process model that satisfies this previously proposed contention. We also contribute to the discussion of DMN function by providing tentative evidence that variation of the gray‐matter volume in DMN regions is linked to the reward circuitry (Figure 2), thus linking two literatures that currently have scarce cross‐references. Finally, we derive explicit hypotheses that could be tested in targeted neuroscience experiments in the future, and we detail how our process model relates to previous cognitive and theoretical accounts of DMN function.

Please appreciate the importance of differentiating which levels of observation are at play in the present account. A process model is not solely intended to capture behavior of the agent, such as cognitive accounts of DMN function, but also the neurocomputational specifics of the agent. Henceforth, we will use “inference” when referring to aspects of the statistical model, “prediction” when referring to the neurobiological implementation, and words like “forecast” or “forsee” when referring to the cognitive behavior of the agent. It is moreover important to note that our account does not claim that neural activity in the DMN in particular or the brain in general are identical with RL algorithms. Rather, we advocate feedback‐based learning strategies as an attractive alternative perspective to describe, quantify, and interpret research findings related to the DMN.

## 3 KNOWN NEUROBIOLOGICAL PROPERTIES OF THE DEFAULT MODE NETWORK

We begin by a neurobiological deconstruction of the DMN based on integrating experimental findings in the neuroscience literature from different species. This walkthrough across main functional zones of the DMN (i.e., de‐emphasizing their precise anatomical properties) will outline the individual functional profiles with the goal of paving the way for their algorithmic interpretation in our formal account (Section 3). We here focus on major *functional* zones of the DMN. Please see elsewhere for excellent surveys on their *anatomical* boundaries and which brain parts could or should be counted as DMN (Binder et al., 2009; Buckner & DiNicola, 2019; Kernbach et al., 2018; Seghier, 2013).

### 3.1 The posteromedial cortex: Global monitoring and information integration

The midline structures of the human DMN, including the posteromedial cortex (PMC) and the medial prefrontal cortex (mPFC), are probably responsible for highest turn‐overs of energy consumption (Gusnard & Raichle, 2001; Raichle et al., 2001). These metabolic characteristics go hand‐in‐hand with brain‐imaging findings that suggested the PMC and mPFC to potentially represent the functional core of the DMN (Andrews‐Hanna, Reidler, Sepulcre, Poulin, & Buckner, 2010; Hagmann et al., 2008).

Normal and disturbed metabolic fluctuations in the human PMC have been closely related to changes of conscious awareness (Cavanna & Trimble, 2006; Leech & Sharp, 2014). Indeed, the PMC matures relatively late (i.e., myelination) during postnatal development in monkeys (Goldman‐Rakic, 1987), which is generally considered to be a sign of evolutionary sophistication. This DMN region has long been speculated to reflect constant computation of environmental statistics and its internal representation as an inner “mind's eye” (Cavanna & Trimble, 2006; Leech & Sharp, 2014). For instance, Bálint's syndrome is a neurological disorder of conscious awareness that can result from tissue damage in the posterior medial cortex (Bálint et al., 1909; Buckner et al., 2008). Such neurological patients are plagued by an inability to bind various individual features of the visual environment into an integrated whole (i.e., simultanagnosia) as well as an inability to direct action toward currently unattended environmental objects (i.e., optic ataxia). Scanning complex scenes is impaired in that statistic or moving objects in the environment may be invisible or disappear in the subject perception of the patient (Blumenfeld, 2002; Mesulam, 2000). This dysfunction can be viewed as a high‐level impairment in gathering information about alternative objects (i.e., exploration) as well as using these environmental opportunities toward a behavioral goal (i.e., exploitation). Congruently, the human PMC was coupled in two different functional connectivity analyses (Bzdok et al., 2015) with the amygdala, involved in significance evaluation, and the nucleus accumbens (NAc), involved in reward evaluation. Specifically, among all parts of the PMC, the ventral posterior cingulate cortex was most connected to the laterobasal nuclei group of the amygdala (Bzdok et al., 2015). This amygdalar subregion has been proposed to continuously scan environmental input for biological relevance assessment (Baxter & Murray, 2002; Bzdok, Laird, Zilles, Fox, & Eickhoff, 2013; Ghods‐Sharifi, Onge, & Floresco, 2009).

The putative role of the PMC in continuous abstract integration of environmental relevance and ensuing top‐level guidance of action on the environment is supported by many neuroscience experiments (Acikalin, Gorgolewski, & Poldrack, 2017; Heilbronner & Platt, 2013). Electrophysiological recordings in animals implicated PMC neurons in strategic decision making (Pearson, Hayden, Raghavachari, & Platt, 2009), risk assessment (McCoy & Platt, 2005), outcome‐dependent behavioral modulation (Hayden, Smith, & Platt, 2009), as well as approach‐avoidance behavior (Vann, Aggleton, & Maguire, 2009). Neuron spiking activity in the PMC allowed distinguishing whether a monkey would pursue an exploratory or exploitative behavioral strategy during food foraging (Pearson et al., 2009). Monkeys were shown to correctly assess the amount of riskiness and ambiguity implicated by behavioral decisions, similar to humans (Hayden, Heilbronner, & Platt, 2010). Further, single‐cell recordings in the monkey PMC demonstrated this brain region's sensitivity to subjective target utility (McCoy & Platt, 2005) and integration across individual decision‐making instances (Pearson et al., 2009). This DMN region encoded the preference for or aversion to options with uncertain reward outcomes and its neural spiking activity was more associated with subjectively perceived relevance of a chosen object than by its actual value, based on an “internal currency of value” (McCoy & Platt, 2005). In fact, direct stimulation of PMC neurons in monkeys promoted exploratory actions, which would otherwise be shunned (Hayden, Nair, McCoy, & Platt, 2008). Graded changes in firing rates of PMC neurons indicated changes in upcoming choice trials, while their neural patterns were distinct from neuronal spike firings that indicated choosing either option. Similarly in humans, the DMN has been shown to gather and integrate information over different parts of auditory narratives in an fMRI study (Simony et al., 2016).

Moreover, the retrosplenial portion of the PMC could support representation of action possibilities and evaluation of reward outcomes by integrating information from memory recall and different perspective frames. Regarding memory recall, retrosplenial damage has been consistently associated with anterograde and retrograde memory impairments of various kinds of sensory information in animals and humans (Vann et al., 2009). Regarding perspective frames, the retrosplenial subregion of the PMC has been proposed to mediate between the organism's egocentric (i.e., focused on external sensory environment) and allocentric (i.e., focused on internal world knowledge) viewpoints in animals and humans (Burgess, 2008; Epstein, 2008; Valiquette & McNamara, 2007).

Consequently, the PMC may contribute to overall DMN function by monitoring the subjective outcomes (Acikalin et al., 2017) of possible actions and integrating that information with memory and perspective frames into short‐ and longer‐term behavioral agendas (Heilbronner & Platt, 2013). Rather than merely detecting novelty (Cooper & Knutson, 2008; Litt, Plassmann, Shiv, & Rangel, 2011), the PMC of the DMN probably represents subjective value for enriching the statistical assessment of the environment to map and predict delayed reward opportunities in the future. Viewed from a RL perspective, the PMC may continuously adapt the organism to changes in both the external environment and its internal representation to enable strategic behavior.

### 3.2 The prefrontal cortex: Action consideration and stimulus‐value association

Analogous to the PMC, the dorsomedial PFC (dmPFC, related to BA9) of the DMN is believed to subserve multi‐sensory processes across time, space, and types of information processing to exert top‐level control on behavior. Comparing to the PMC, however, dmPFC function may be closer to a “mental sketchpad” (Goldman‐Rakic, Cools, & Srivastava, 1996). This DMN part potentially subserves the de‐novo construction and manipulation of meaning representations instructed by stored semantics and memories (Bzdok et al., 2013; Eickhoff et al., 2016). The dmPFC may subserve representation and assessment of one's own and other individuals' action considerations—a necessary component of a full‐blown RL agent. Generally, neurological patients with tissue damage in the prefrontal cortex are known to struggle with adaptation to new stimuli and events (Stuss & Benson, 1986). Specifically, neural activity in the human dmPFC reflected expectations about other peoples' actions and outcomes of these predictions. Neural activity in the dmPFC indeed explained the performance decline of inferring other peoples' thoughts in aging humans (Moran, Jolly, & Mitchell, 2012). Certain dmPFC neurons in macaque monkeys exhibited a preference for processing others', rather than own, action with fine‐grained adjustment of contextual aspects (Yoshida, Seymour, Friston, & Dolan, 2010).

**FIGURE 1:**
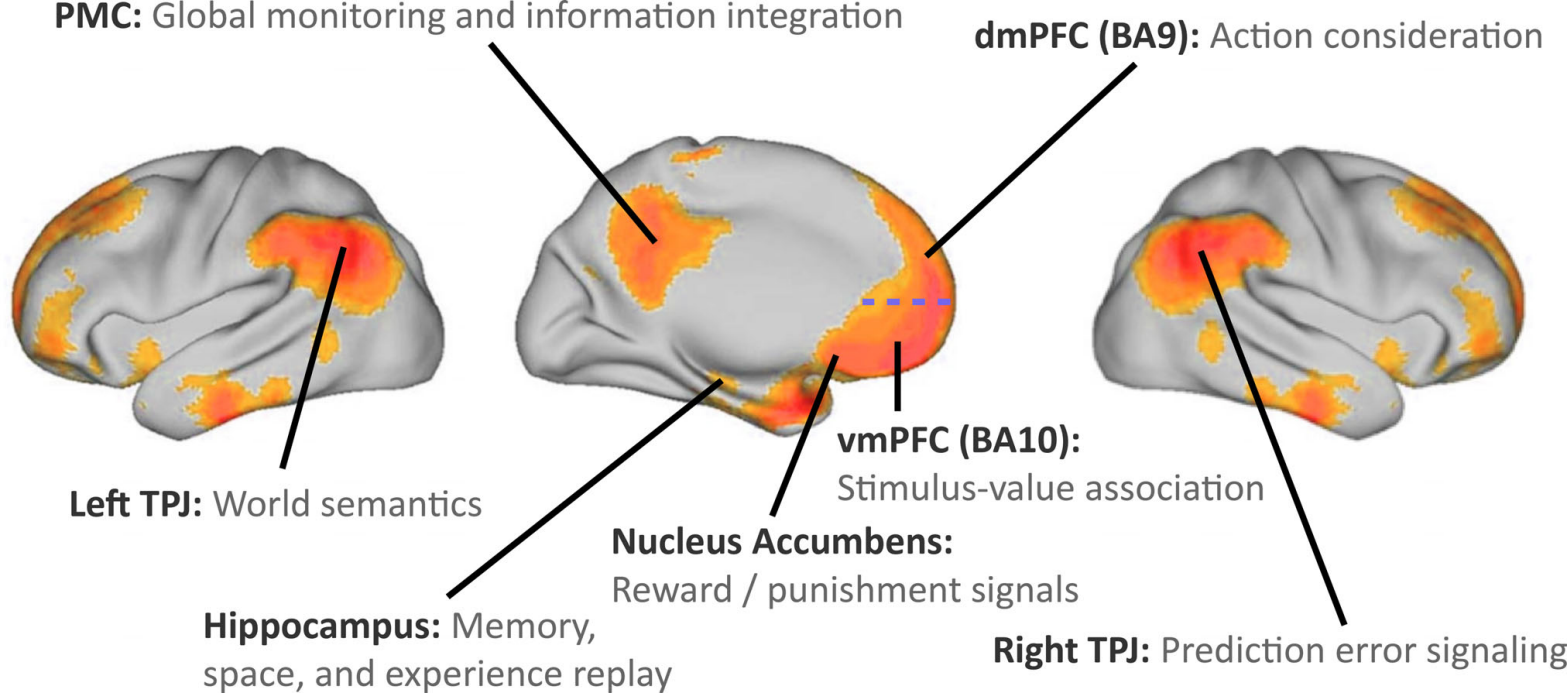
Default mode network: key functions. Neurobiological overview of the DMN with its major constituent parts and the associated functional roles relevant in our functional interpretation. The blue horizontal dashed line indicates the cytoarchitectonic border between the more dorsal BA9 and the more ventral BA10 (Brodmann, 1909). Axonal tracing in monkeys and diffusion tractography in humans suggested that the NAc of the reward circuitry has monosynaptic fiber connections to the vmPFC (Croxson et al., 2005; Haber, Kunishio, Mizobuchi, & Lynd‐Balta, 1995b). Evaluation of propagated value information and triggered affective states encoded in the vmPFC may then feed into the functionally connected partner nodes of the DMN, such as the dmPFC and PMC (Andrews‐Hanna et al., 2010; Bzdok, Langner, Schilbach, Engemann, et al., 2013)

**FIGURE 2:**
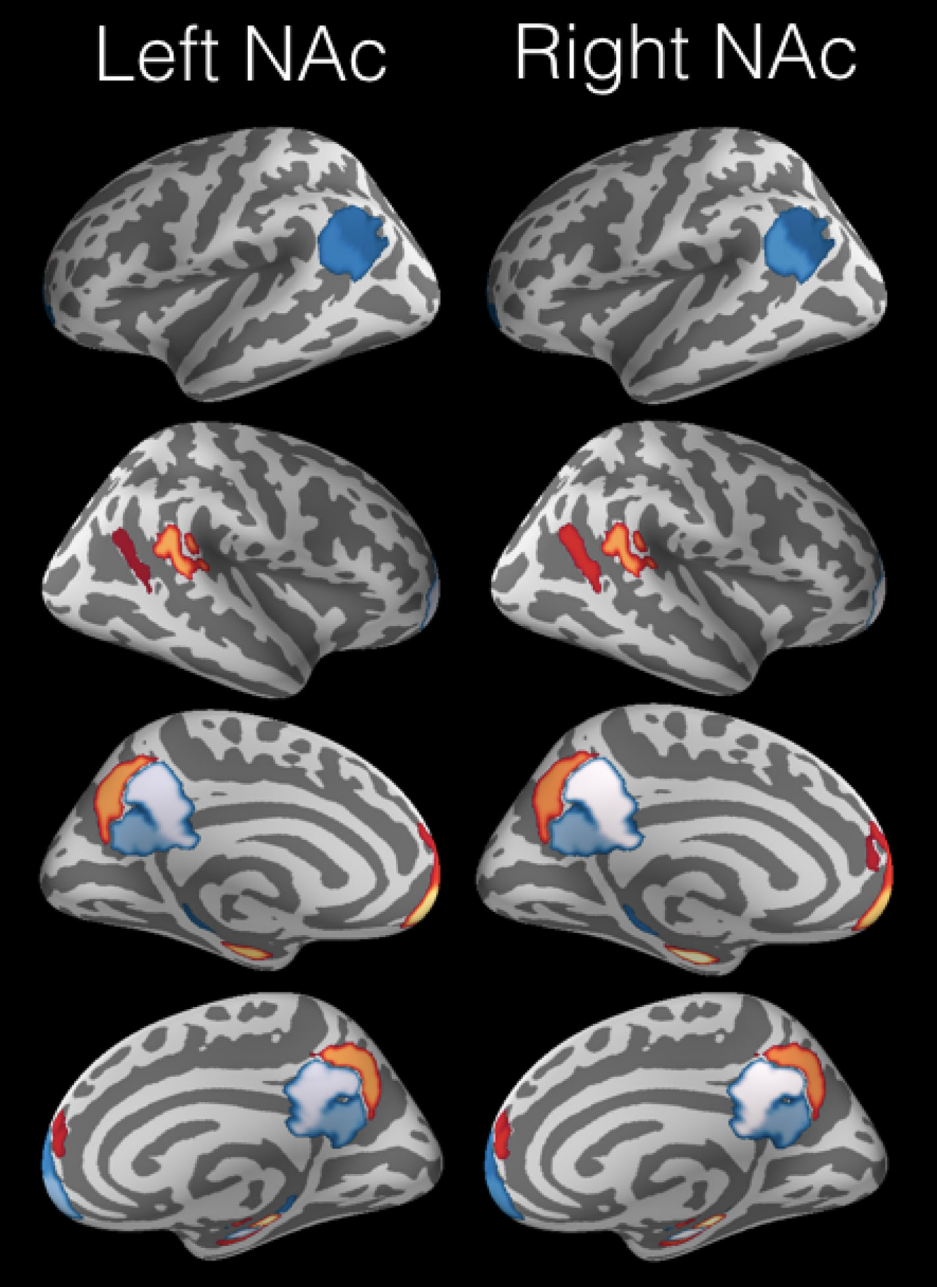
Predictive structural association between reward system and DMN nodes. Reward tasks (O'Doherty, Lee, & McNamee, 2015) and neural processing in the DMN (Buckner et al., 2008), often considered “task‐negative,” have been studied so far in largely separate niches of the neuroscience literature. A currently underappreciated link is however suggested here based on 9,932 human subjects from the UK Biobank, inter‐individual differences in left NAc volume (*R* ^2^ = 0.11 ± 0.02 [standard deviation across cross‐validation folds]) and right NAc volume (*R* ^2^ = 0.14 ± 0.02) could be predicted from (*z*‐scored) volume in the DMN regions. These out‐of‐sample generalizations reflect the expected performance in yet‐to‐be observed individuals (Bzdok & Ioannidis, 2019) obtained from linear support vector regression applied to region volume measures in the DMN in a 10‐fold cross‐validation procedure (Hastie, Tibshirani, & Friedman, 2011). Consistent for the left and right reward system, NAc volume in a given subject is positively coupled with the vmPFC and HC. The congruence of our structural association results for both NAc targets speaks to the robustness of our pattern‐prediction findings. The opposite relation of the left and right TPJ to the NAc appears to reflect a repeatedly recognized hemispheric asymmetry with respect to functional implications (Seghier, 2013), impairments in neurological patients (Corbetta, Kincade, Ollinger, McAvoy, & Shulman, 2000), different types of connectivity (Caspers et al., 2011; Uddin et al., 2010) as well as micro‐ and macroanatomy (Caspers et al., 2006, 2008). The colors are indicative of the (red = positive, blue = negative) and relative importance (the lighter the higher) of the regression coefficients. The code for reproduction and visualization: www.github.com/banilo/darkcontrol_2018

Comparing to the dmPFC, the ventromedial PFC (vmPFC, related to BA10) is probably more specifically devoted to subjective value evaluation and risk estimation of relevant environmental stimuli (Figures 1 and 2). The ventromedial prefrontal DMN may subserve adaptive behavior by bottom‐up‐driven processing of “what matters now,” drawing on sophisticated value representations (Kringelbach & Rolls, 2004; O'Doherty et al., 2015). Quantitative lesion findings across 344 human individuals confirmed a substantial impairment in value‐based action choice (Gläscher et al., 2012). Indeed, this DMN region is preferentially connected with reward‐related and limbic regions. The vmPFC is well known to have direct connections with the NAc in axonal tracing studies in monkeys (Haber, Kunishio, Mizobuchi, & Lynd‐Balta, 1995). Congruently, the gray‐matter volume of the vmPFC and NAc correlated with indices of value‐guided behavior and reward attitudes in humans (Lebreton, Jorge, Michel, Thirion, & Pessiglione, 2009). NAc activity is further thought to reflect reward prediction signals from dopaminergic neurotransmitter pathways (Schultz, 1998) that not only channel action toward basic survival needs, but also enable more abstract reward processings, and thus perhaps RL, in humans (O'Doherty et al., 2015).

Consistently, diffusion MRI tractography in humans and monkeys (Croxson et al., 2005) quantified the NAc to be more connected to the vmPFC than dmPFC in both species. Two different functional connectivity analyses in humans also revealed strong vmPFC connections with the NAc, hippocampus (HC), and PMC (Bzdok et al., 2015). In line with these connectivity findings in animals and humans, the vmPFC is often proposed to represent triggered emotional and motivational states (Damasio, Everitt, & Bishop, 1996). Such real or imagined arousal states could be mapped in the vmPFC as a bioregulatory disposition influencing cognition and decision making. In neuroeconomic studies of human decision making, the vmPFC consistently reflects an individual's subjective value predictions (Behrens, Hunt, Woolrich, & Rushworth, 2008). This finding may also explain why performance within and across participants was reported to relate to state encoding in the vmPFC (Schuck, Cai, Wilson, & Niv, 2016). Such a “cognitive map” of the action space—an integral part of a RL agent—was argued to encode the current task state even when states are unobservable from the sensory environment.

### 3.3 The hippocampus: Memory, space, and experience replay

The DMN midline has close functional links with the HC (henceforth implying to include also parahippocampal regions) in the medial temporal lobe (Shannon et al., 2013; Vincent et al., 2006)—a region long known to be involved in memory operations and spatial navigation in animals and humans. While the HC is traditionally believed to allow recalling past experience, there is now increasing evidence for an important role in constructing mental models in general (Boyer, 2008; Gelbard‐Sagiv, Mukamel, Harel, Malach, & Fried, 2008; Javadi et al., 2017; Schacter et al., 2007; Zeidman & Maguire, 2016). Its recursive anatomical architecture may be specifically designed to allow reconstructing entire sequences of experience from memory fragments. Indeed, hippocampal damage was not only associated with an impairment in re‐experiencing the past (i.e., amnesia), but also forecasting of one's own future and imagination of experiences more broadly (Hassabis et al., 2007).

Mental scenes created by neurological patients with HC lesion exposed a lack of spatial integrity, richness in detail, and overall coherence (c.f, Hassabis et al., 2007). Single‐cell recordings in the animal HC revealed constantly active neuronal populations whose firing coincided with specific locations in space during environmental navigation. Indeed, when an animal is choosing between alternative paths, the corresponding neuronal populations in the HC spike one after another (Johnson & Redish, 2007). Such neuronal patterns in the HC appear to directly indicate upcoming behavior, such as in planning navigational trajectories (Pfeiffer & Foster, 2013) and memory consolidation of choice relevance (De Lavilléon, Lacroix, Rondi‐Reig, & Benchenane, 2015). Congruently, London taxi drivers, humans with high performance in forecasting spatial navigation, were shown to exhibit increased gray‐matter volume in the HC (Maguire et al., 2000).

There is hence increasing evidence that HC function extends beyond simple forms of encoding and reconstruction of memory and space information. Based on spike recordings of hippocampal neuronal populations, complex spiking patterns can be followed across extended periods including their modification of input‐free self‐generated patterns after environmental events (Buzsáki, 2004). Specific spiking sequences, which were elicited by experimental task design, have been shown to be re‐enacted spontaneously during quiet wakefulness and sleep (Hartley, Lever, Burgess, & O'Keefe, 2014; O'Neill, Pleydell‐Bouverie, Dupret, & Csicsvari, 2010). Moreover, neuronal spike sequences measured in hippocampal place cells of rats featured reoccurrence directly after experimental trials as well as directly before (prediction of) upcoming experimental trials (Diba & Buzsáki, 2007). Similar spiking patterns in hippocampal neurons during rest and sleep have been proposed to be critical in communicating local information to the neocortex for long‐term storage, potentially including DMN regions. Moreover, in mice, invasively triggering spatial experience recall in the HC during sleep has been demonstrated to subsequently alter action choice during wakefulness (De Lavilléon et al., 2015). These HC‐subserved mechanisms conceivably contribute to advanced cognitive processes that require re‐experiencing or newly constructed mental scenarios, such as in recalling autobiographical memory episodes (Hassabis et al., 2007). Within a RL framework, the HC could thus orchestrate re‐experience of environmental aspects for consolidations based on re‐enactment and for integration into rich mental scene construction (Bird, Capponi, King, Doeller, & Burgess, 2010; Deuker, Bellmund, Schröder, & Doeller, 2016). In this way, the HC may impact ongoing perception of and action on the environment (De Lavilléon et al., 2015; Zeidman & Maguire, 2016).

### 3.4 The right and left TPJ: Prediction error signaling and world semantics

The DMN emerges with its midline structures early in human development (Doria et al., 2010), while the right and left TPJs may become fully functionally integrated into this macroscopical network only after birth. The TPJs are known to exhibit hemispheric differences based on microanatomical properties and cortical gyrification patterns (Seghier, 2013). In general, neuroscientific investigations on hemispheric functional specialization have highlighted the right cerebral hemisphere as more dominant for attentional functions and the left side more for semantic functions (Bzdok et al., 2013, 2016; Seghier, 2013; Stephan, Fink, & Marshall, 2007).

The TPJ in the right hemisphere (RTPJ) denotes a broad functional zone with varying anatomical nomenclature (Mars et al., 2011; Seghier, 2013; Seghier, Fagan, & Price, 2010) that has been shown to be closely related to multi‐sensory event representation and prediction error signaling (Downar, Crawley, Mikulis, & Davis, 2000; Vetter, Butterworth, & Bahrami, 2011; Shulman et al., 2010; Shulman, Astafiev, McAvoy, d'Avossa, & Corbetta, 2007). This DMN region is probably central for action initiation during goal‐directed psychological tasks and for sensorimotor behavior by integrating multi‐sensory attention (Corbetta & Shulman, 2002). Its involvement was repeatedly reported in monitoring multi‐step action execution (Hartmann, Goldenberg, Daumüller, & Hermsdörfer, 2005), visuo‐proprioceptive conflict (Balslev, Nielsen, Paulson, & Law, 2005), spatial reorientation (Corbetta et al., 2000), and detection of environmental changes across visual, auditory, or tactile stimulation (Downar et al., 2000). Direct electrical stimulation of the human RTPJ during neurosurgery was associated with altered perception and stimulus awareness (Blanke, Ortigue, Landis, & Seeck, 2002). It was argued that the RTPJ encodes actions and predicted outcomes, without necessarily relating these neural processes to value estimation (Hamilton & Grafton, 2008; Jakobs et al., 2009; Liljeholm, Wang, Zhang, & O'Doherty, 2013; Rutledge et al., 2009). More specifically, neural activity in the RTPJ has been proposed to reflect stimulus‐driven attentional reallocation to self‐relevant and unexpected sources of information as a “circuit breaker” that recalibrates functional control of brain networks (Bzdok, Langner, Schilbach, Jakobs, et al., 2013; Corbetta, Patel, & Shulman, 2008). In the face of large discrepancies between actual and previously predicted environmental events, the RTPJ may act as a potential switch between externally‐oriented mind sets focussed on the sensory environment and internally‐oriented mind sets focussed on mental scene construction. For instance, temporally induced RTPJ damage in humans diminished the impact of predicted intentions of other individuals (Young, Camprodon, Hauser, Pascual‐Leone, & Saxe, 2010), a capacity believed to be enabled by the DMN. Viewed from a RL perspective, the RTPJ might reflect an important relay that shifts away from the “internally directed” baseline processes to, instead, deal with unexpected environmental cues and events.

The left TPJ of the DMN (LTPJ), in turn, may have a functional relationship to Wernicke's area involved in semantic processes (Blumenfeld, 2002) and has been described as “a temporoparietal transmodal gateway for language” by some investigators (Mesulam, 2000). Neurological patients with damage in this region have a major impairment of language comprehension when listening to others or reading a book. Patient speech preserves natural rhythm and normal syntax, yet the voiced sentences lack meaning (i.e., aphasia). Abstracting from speech interpretations in linguistics and neuropsychology, the LTPJ appears to mediate access to and binding of world knowledge, such as required during action considerations (Binder & Desai, 2011; Seghier, 2013). Consistent with this view, LTPJ damage in humans also entailed problems in recognizing others' pantomimed action toward objects without obvious relation to processing explicit language content (Varney & Damasio, 1987).

Inner speech also hinges on knowledge recall about the physical and social world. Indeed, the internal production of verbalized thought (“language of the mind”) was closely related to the LTPJ in a pattern analysis of brain volume (Geva et al., 2011). Further, episodic memory recall and mental imagery to forecast future events strongly draw on reassembling world knowledge. Isolated building blocks of world structure get rebuilt in internally constructed mental scenarios that guide present action choice, weigh hypothetical possibilities, and forecast event outcomes. As a candidate component of a RL agent, neural processes in the LTPJ may contribute to the automated predictions of the environment by incorporating experience‐derived building blocks of world regularities into ongoing action, planning, and problem solving.

## 4 RL CONTROL: A PROCESS MODEL FOR DMN FUNCTION

We argue the outlined neurobiological properties of the DMN regions to be sufficient for implementing all components of a full‐fledged RL system. Recalling past experience, considering candidate actions, random sampling of possible experiences, as well as estimation of instantaneous and delayed reward outcomes are key components of intelligent RL agents that are plausible to functionally intersect in the DMN.

**FIGURE 3:**
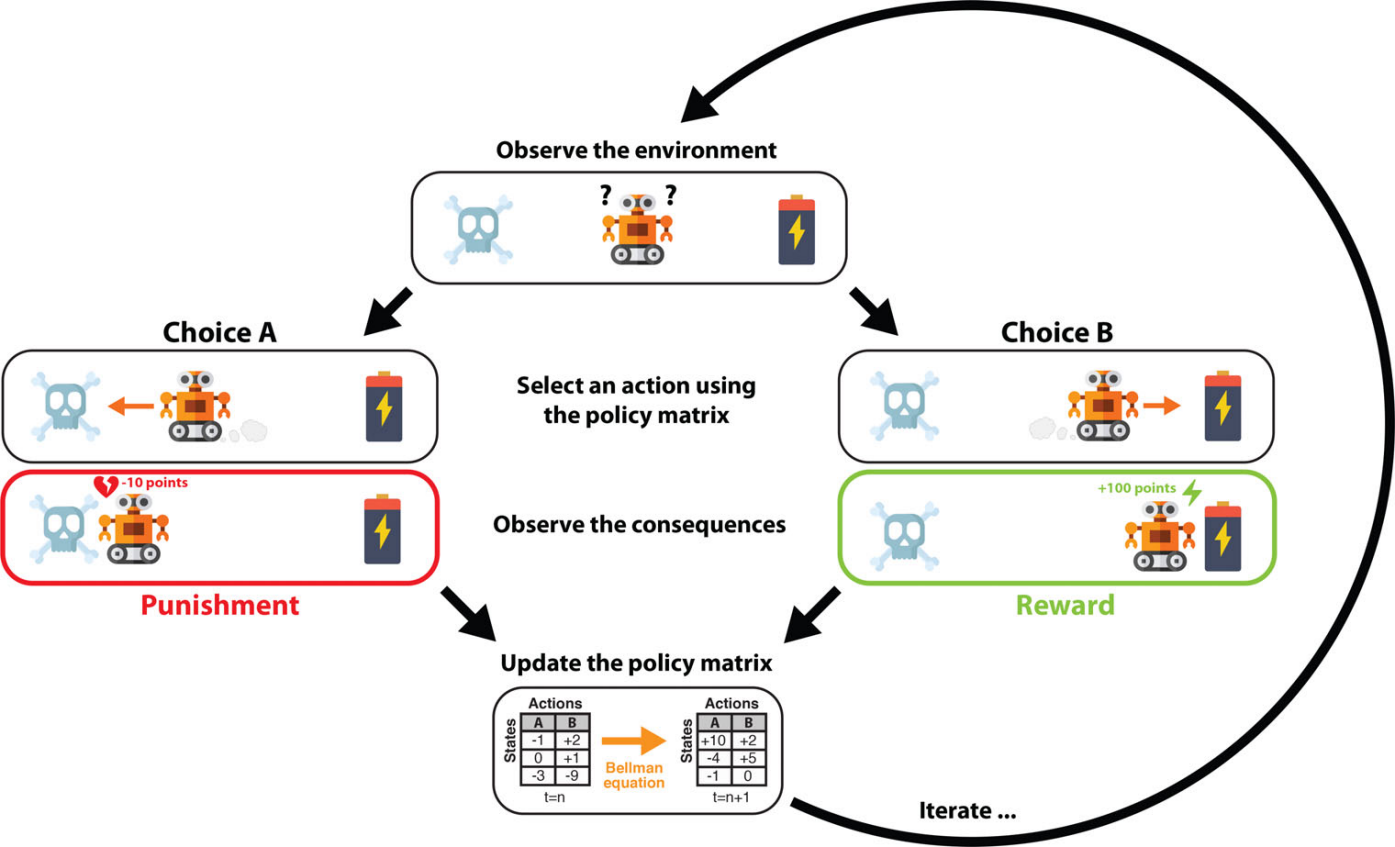
Illustration of a partially observable Markov decision process (POMDP). Given the current state of the environment, the agent takes an action by following the policy matrix, which is iteratively updated by the Bellman equation. The agent receives a triggered reward and observes the next state. The process goes on until interrupted or a goal state is reached

RL is an area of machine learning concerned with searching optimal behavioral strategies through interactions with an *environment* with the goal to maximize the *cumulative reward* over time (Sutton & Barto, 1998). Optimal behavior typically takes the future into account as certain rewards could be *delayed*. Through repeated action on and feedback from the environment, the agent learns how to reach goals and continuously improve the collection of reward signals in a trial‐and‐error fashion (Figure 3). At a given moment, each taken *action a* triggers a change in the *state* of the environment *s* → *s*^′^, accompanied by environmental feedback signals as *reward r* = *r*(*s*, *a*, *s*^′^) obtained by the agent. If the collected reward outcome yields a negative value it can be more naturally interpreted as *punishment*. The environment can be partly controlled by the action of the agent and the reward can be thought of as satisfaction—or aversion—that accompany the execution of a particular action.

The environment is assumed to be *stochastic*, that is, changing in random ways. In addition, the environment is only *partially observable* in the sense that only limited aspects of the environment's state are accessible to the agent's sensory perception (Starkweather, Babayan, Uchida, & Gershman, 2017). We assume that volatility of the environment is realistic in a computational model which sets out to explain DMN functions of the human brain.

We argue that an abstract description of DMN activity based on RL can naturally embed human behavior in the unavoidable tradeoff between exploitative action with immediate gains and explorative action with longer‐term reward outcomes (Dayan & Daw, 2008). In short, DMN implication in a diversity of particularly sophisticated human behaviors can be parsimoniously explained as instantiating probabilistic simulations of experience coupled with prediction error minimization to calibrate action trajectories for reward outcome maximization at different time scales. Such a purposeful optimization objective may be subserved by a stochastic approximation based on a brain implementation for Monte Carlo sampling of events and outcomes.

### 4.1 Markov decision processes

In artificial intelligence and machine learning, a popular computational model for multi‐step decision processes are MDPs (Sutton & Barto, 1998). An MDP operationalizes a sequential decision process in which it is assumed that environment dynamics are determined by a Markov process, but the agent cannot directly observe the underlying state. Instead, the agent tries to optimize a *subjective* reward signal (i.e., likely to be different for another agent in the same state and possibly driven by neural processing in the vmPFC) by maintaining probability distributions over actions (possibly represented in the dmPFC) according to their expected utility.

This is a minimal set of assumptions that can be made about an environment faced by an agent engaged in interactive learning.

Definition. Mathematically, an MDP involves a quadruple $\left(S,A,r,p\right)$ where.

- S is the set of states, such as $S=\left\{\text{happy},\mathrm{sad},\text{puzzeled}\right\}$.
- A is the set of actions, such as $A=\left\{\text{read},,,\mathrm{run},,,\text{laugh},,,\text{sympathize},,,\text{empathize}\right\}.$
- r:S×A×S→ℝ is the *reward function*, so that *r*(*s*, *a*, *s*′) is the instant reward for taking action *a* in state *s* followed by a state‐transition *s* → *s*^′^.
- $p:S\times A\times S\to \left[0,1\right],\;\left(s,a,s^{\prime }\right)\mapsto p\left(s^{\prime }|s,a\right)$, the probability of moving to state *s*′ if action *a* is taken from state *s*. In addition, one requires that such transitions be Markovian. Consequently, the future states are independent of past states and only depend on the present state and action taken.

The process has *memory* if the subsequent state depends not only on the current state but also on a number of past states. Rational probabilistic planning can thus be reformulated as a standard memoryless Markov process by simply expanding the definition of the state *s* to include experience episodes of the past. This extension adds the capacity for memory to the model because the next state then depends not only on the current situation, but also on previously experienced events, which is the motivation behind partially observable MDPs (POMDPs; O'Reilly & Frank, 2006; Starkweather et al., 2017). Nevertheless, this mathematical property of POMDPs mostly accounts for implicit memory. Since the current article is concerned with plausibility at the behavioral and neurobiological level, we will address below how our account can accommodate the neurophysiological constraints of the DMN and the explicit memory characteristics of human agents.

#### 4.1.1 Why Markov decision processes?

One may wonder whether MDP models are applicable to something as complex as human behavior. This class of reinforcement‐learning models has had numerous successes in diverse applied domains. For instance, MDPs have been successfully used in financial trading is largely a manifestation of strategic decision‐making of interacting human agents. According to how the market responds, the agent incurs gain or loss as environmental feedback of the executed financial actions. Recent research on automatizing market exchanges by algorithmic trading has effectively deployed MDPs as a framework for modeling these elaborate behavioral dynamics (Abergel, Huré, & Pham, 2017; Brázdil, Chatterjee, Forejt, & Kucera, 2017; Dempster & Leemans, 2006; Hult & Kiessling, 2010; Yang et al., 2012; Yang, Qiao, Beling, & Scherer, 2014; Yang, Qiao, Beling, Scherer, & Kirilenko, 2015). MDPs have also been effective as a behavioral model in robotics (Abbeel & Ng, 2004; Ng et al., 2004) and in challenging multistep strategy games (Mnih et al., 2015; Pritzel et al., 2017; Silver et al., 2016). More recent work has developped an MDP‐related way of reasoning about future behavior of other agents (Rabinowitz et al., 2018). The idea is to use meta‐learning (i.e., learning to learn) to build strong priors about the behavior of a population of other agents.

#### 4.1.2 Reinforcement learning in the brain?

RL has been argued to be a biologically plausible mechanism in the human brain (Daw & Dayan, 2014; O'Doherty et al., 2015). Indeed, previous authors have proposed (Gershman, Horvitz, & Tenenbaum, 2015) that a core property of human intelligence is the improvement of expected utility outcomes as a strategy for action choice in uncertain environments, a view captured by the formalism of MDPs. It has also long been proposed (Dayan & Daw, 2008) that there can be a mapping between algorithmic aspects underlying model‐free and model‐based RL and neurobiological aspects underlying decision‐making, which involves parts of the DMN. The neurotransmitter dopamine could serve as a “teaching signal” to guide estimation of value associations and action policies by modulating synaptic plasticity in the reward‐processing circuitry, including the NAc. In contrast, model‐based RL would start off with some mechanistic assumptions about the dynamics of the world. These assumptions could relate to the physical laws governing the agent's environment, constraints on the state space, transition probabilities between states, or reward contingencies.

An agent might represent such knowledge about the world as follows:

- *r*(*s*, “stand still”) = 0 if *s* does not correspond to a location offering relevant resources.
- *p*(*s*′|*s*, “stand still”) = 1 if *s*′ = *s* and 0 otherwise.
- etc.

Such knowledge can be partly extracted from the environment: the agent infers a model of the world while learning to take optimal decisions based on the current representation of the environment. These methods learn what the effect is going to be of taking a particular action in a particular state. The result is an estimate of the underlying MDP which can then be either solved exactly or approximately, depending on the setting and what is feasible.

#### 4.1.3 Accumulated reward and policies

The behavior of the agent is governed by a *policy*, which maps states of the world to probability distributions over candidate actions (potentially represented in the dmPFC). Starting at time *t* = 0, following a policy *π* generates a trajectory of action choices:

$$\begin{array}{l} \text{choose action}:a_{0}\sim \pi \left(a|s_{0}\right)\\ \text{observe transition}:s_{1}\sim p\left(s|s_{0},a_{0}\right)\;\text{and collect reward}\;R_{0}=r\left(s_{0},a_{0},s_{1}\right)\\ \text{choose action}:a_{1}\sim \pi \left(a|s_{1}\right)\\ \text{observe transition}:s_{2}\sim p\left(s|s_{1},a_{1}\right),\text{and collect reward}\;R_{1}=r\left(s_{1},a_{1},s_{2}\right)\\ \vdots \\ \text{choose action}:a_{t}\sim \pi \left(a|s_{t}\right)\\ \text{observe transition}:s_{t+1}\sim p\left(s|s_{t},a_{t}\right),\text{and collect reward}\;R_{t}=r\left(s_{t},a_{t},s_{t+1}\right)\\ \vdots \end{array}$$

We assume time invariance in that we expect the dynamics of the process to be equivalent over sufficiently long time windows of equal length (i.e., stationarity). Since an action executed in the present moment might have repercussions in the far future. It turns out that the quantity to optimize is not the instantaneous rewards *r*(*s*, *a*), but a *cumulative reward* estimate which takes into account expected reward from action choices in the future. A common approach to modeling this gathered outcome, which is likely to involve extended parts of the DMN, is the time‐discounted cumulative reward

(1) $$G^{\pi }=\sum_{t=0}^{\infty }\gamma^{t}R_{t}=R_{0}+\gamma R_{1}+\gamma^{2}R_{2}+\ldots +\gamma^{t}R_{t}+\ldots$$

This random variable measures the cumulative reward of following an action policy *π*. The reward outcome is random because it depends both on the environment's dynamics and the policy *π* being executed. The exponential delay discounting function used here refers to the usual formulation in the field of reinforcement learning, although psychological experiments may also reveal other discounting regimes (Green & Myerson, 2004). Note that value buffering may be realized in the vmPFC by virtue of this region's connections to the NAc of the reward system (Carlezon & Thomas, 2009; Croxson et al., 2005; Haber et al., 1995b).

The goal of the RL agent is then to successively update this action policy (perhaps most closely related to the PMC) in order to maximize *G* ^*π*^ on average (cf., below). In Equation (1), the definition of cumulative reward *G* ^*π*^, the constant *γ* (0 ≤ *γ* < 1) is the *reward discount factor*, viewed to be characteristic trait for a certain agent. On the one hand, setting *γ* = 0 yields perfectly hedonistic behavior. An agent with such a shortsighted time horizon is exclusively concerned with immediate rewards. This is however not compatible with coordinated planning of longer‐term agendas that is potentially subserved by neural activity in the DMN.

On the other hand, setting 0 < *γ* < 1 allows a learning process to arise. A positive *γ* can be seen as calibrating the risk‐seeking trait of the intelligent agent, that is, the behavioral predispositions related to trading longer delays for higher reward outcomes. Such an agent puts relatively more emphasis on rewards expected in a more distant future. Concretely, rewards that are not expected to occur only within a very large number of time steps from the present point are ignored. The complexity reduction by time discounting alleviates the variance of expected rewards accumulated across considered action cascades by limiting the depth of the search tree. Given that there is more uncertainty in the far future, it is important to appreciate that a stochastic policy estimation is more advantageous in many RL settings.

### 4.2 The components of reinforcement learning in the DMN

Given only the limited information available from an MDP, at a state *s* the average utility of choosing an action *a* under a policy *π* can be captured by the single quantity

(2) $$Q^{\pi }\left(s,a\right)=E\left[G^{\pi }|s_{0}=s,a_{0}=a\right],$$

called the *Q*‐value for the state‐action pair (*s*, *a*). In other words, *Q* ^*π*^(*s*, *a*) corresponds to the expected reward over all considered action trajectories, in which the agent sets out in the environment in state *s*, chooses action *a*, and then follows the policy *π* to select future actions.

For the brain, *Q* ^*π*^(*s*, *a*) defined in Equation (2) provides the subjective utility of executing a specific action. In this way, we can answer the question “What is the expected utility of choosing action *a*, and its ramifications, in this situation?.” *Q* ^*π*^(*s*, *a*) offers a formalization of optimal behavior that may well capture processing aspects such as subserved by the DMN in human agents.

#### 4.2.1 Optimal behavior and the Bellman equation

Optimal behavior of the agent corresponds to a strategy π^*^ for choosing actions such that, for every state, the chosen action guarantees the best possible reward on average. Formally,

(3) $$\pi^{*}\left(s\right)≔\arg\;\max_{a\in A}Q^{*}\left(s,a\right),\text{where}\;Q^{*}\left(s,a\right)≔\max_{\pi }Q^{\pi }\left(s,a\right).$$

The learning goal is to approach the ideal policy π^*^ as close as possible, that is, to solve the MDP. Note that Equation (3) presents merely a definition and does not lend itself as a candidate schema for fully computing MDPs with even moderately sized action and state spaces (i.e., computational intractability).

Fortunately, the *Bellman equation* (Sutton & Barto, 1998) provides a fixed‐point relation which defines Q^*^ implicitly via a sampling procedure, without querying the entire space of policies, with the form

(4) $$Q^{*}=\mathrm{Bel}\left(Q^{*}\right),$$

where the so‐called Bellman transform Bel(*Q*) of an arbitrary *Q*‐value function Q:S×A→ℝ is another *Q*‐value function defined by

(5) $$\begin{array}{l} \mathrm{Bel}\left(Q\right)\left(s,a\right)≔E_{s^{\prime }\sim p\left(s^{\prime }|s,a\right)}\left[r\left(s,a\right)+\gamma \max_{a^{\prime }\in A}Q\left(s^{\prime },a^{\prime }\right)\right]\\ =r\left(s,a\right)+\gamma E_{s^{\prime }\sim p\left(s^{\prime }|s,a\right)}\left[\max_{a^{\prime }\in A}Q\left(s^{\prime },a^{\prime }\right)\right]\\ =\text{instantaneous reward}+\text{expected reward for acting greedily thereafter} \end{array}$$

The Bellman Equation (4) is a temporal consistency equation which provides a dynamic decomposition of optimal behavior by dividing the *Q*‐value function into the immediate reward component and the discounted reward component of the upcoming states. The optimal Q‐value operator Q^*^ is a fixed point for this equation. As a consequence of this outcome stratification, the complicated dynamic programming problem (3) is broken down into simpler sub‐problems at different time points. Indeed, exploitation of hierarchical structure in action considerations has previously been related to the medial prefrontal part of the DMN (Braver & Bongiolatti, 2002; Koechlin, Basso, Pietrini, Panzer, & Grafman, 1999). Using the Bellman equation, each state can be associated with a certain value to guide action toward a preferred state, thus improving on the current action policy of the agent.

Note that in Equation (4) the random sampling is performed only over quantities which depend on the environment. This aspect of the learning process can unroll off‐policy by observing state transitions triggered by another (possibly stochastic) behavioral policy.

#### 4.2.2 Value approximation and the policy matrix

As already mentioned in the previous section, Q‐learning (Watkins & Dayan, 1992) optimizes over the class of deterministic policies of the form Equation (3). State spaces may be extremely large and tracking all possible states and actions may require prohibitively excessive computation and memory resources, perhaps reflect in the especially high metabolic turn‐over of the posterior medial DMN (i.e., PMC). The need of maintaining an explicit table of states can be eliminated by instead using of an approximate *Q*‐value function $\tilde{Q}\left(s,a|\theta \right)$ by keeping track of an approximating parameter *θ* of much lower dimension than the number of states. At a given time step, the world is in a state s∈S, and the agent takes an action which it expects to be the most valuable on average, namely,

(6) $$\pi^{\text{hard}‐\max}\left(s\right)=\arg\;\max_{a\in A}\tilde{Q}\left(s,a|\theta \right).$$

This defines a mapping from states directly to actions.

For instance, a simple linear model with a kernel *ϕ* would be of the form $\tilde{Q}\left(s,a|\theta \right)=\phi {\left(s,a\right)}^{T}\theta$, where *ϕ*(*s*, *a*) would represent a high‐level representation of the state‐action pairs (*s*, *a*), as was previously proposed (Song, Parr, Liao, & Carin, 2016), or artificial neural‐network models as demonstrated in seminal machine‐learning models (Mnih et al., 2015; Silver et al., 2016) for playing complex games (atari, Go, etc.) at super‐human levels.

In the DMN, the dmPFC is conceivable to implement such a hard‐max lookup over the action space. The model parameters *θ* would correspond to synaptic weights and connection strengths within and between brain regions. It is a time‐varying neuronal program which dictates how to move from world states *s* to actions *a* via the hard‐max policy Equation (6). The approximating *Q*‐value function $\tilde{Q}\left(s,a|\theta \right)$ would inform the DMN with the (expected) usefulness of choosing an action *a* in state *s*. The DMN, and in particular its dmPFC part, could then contribute to the choice, at a given state *s*, of an action *a* which maximizes the approximate *Q*‐values. This mapping from states to actions is conventionally called *policy matrix* (Mnih et al., 2015; Silver et al., 2016). Learning consists in starting from a given table and updating it during action choices, potentially reflected in neural processing in the PMC, which take the agent to different table entries.

#### 4.2.3 Self‐training and the loss function

Successful learning in brains and computer algorithms may not be possible without a defined optimization goal—the *loss function*. The action *a* chosen in state *s* according to the policy matrix defined in Equation (6) yields a reward *r* collected by the agent, after which the environment transitions to a new state $s^{\prime }\in S$. One such cycle yields a new *experience e* = (*s*, *a*, *r*, *s′*). Each cycle represents a behavior unit of the agent and is recorded in replay memory buffer—which we hypothesize to involve especially the HC—, possibly discarding the oldest entries to make space: $D\leftarrow \text{append}\left(D,e\right)$. At time step *k*, the agent seeks an update *θ*_*k*_ ← *θ*_*k* − 1_ + *δθ*_*k*_ of the parameters for its approximate model of the *Q*‐value function. Step‐by‐step model parameter updates warrant a learning process and definition of a loss function. The Bellman Equation (4) provides a way to obtain such a loss function (9) as we outline in the following.

**FIGURE 4:**
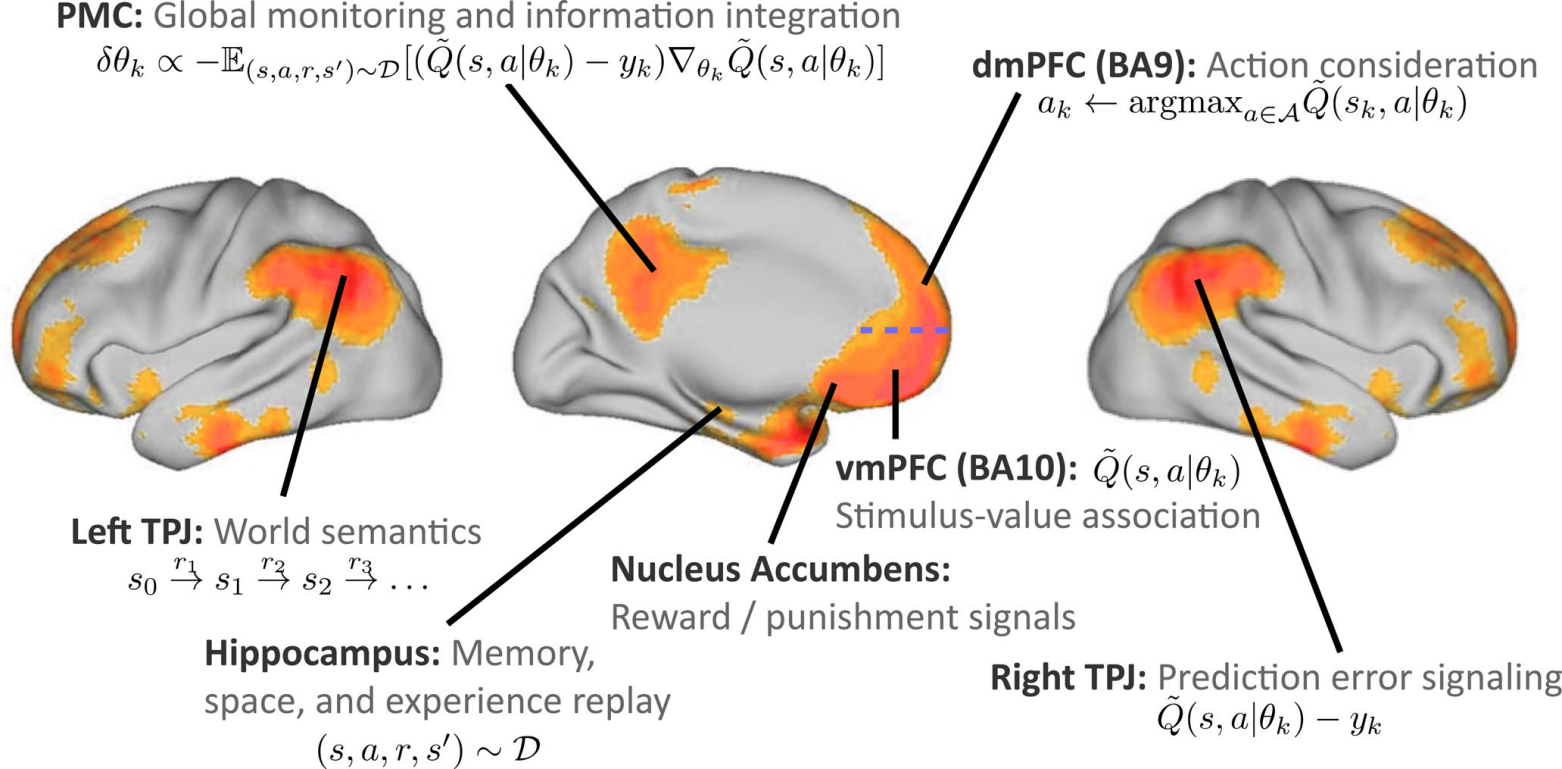
Default mode network: possible neurobiological implementation of reinforcement learning. Overview of how the constituent regions of the DMN (refer to Section 3; blue horizontal dashed line indicates the border between BA9 and BA10) may map onto computational components necessary for a RL agent. Axonal tracing in monkeys and diffusion tractography in humans suggested that the NAc of the reward circuitry has monosynaptic fiber connections to the vmPFC (Croxson et al., 2005; Haber et al., 1995b). Evaluation of propagated value information and triggered affective states encoded in the vmPFC may then feed into the functionally connected partner nodes of the DMN, such as the dmPFC and PMC (Andrews‐Hanna et al., 2010; Bzdok, Langner, Schilbach, Engemann, et al., 2013)

Experience replay consists in sampling batches of experiences *e* $\left(s,a,r,s^{\prime }\right)\sim D$ from the replay memory D. The agent then tries to approximate the would‐be *Q*‐value for the state‐action pair (*s*, *a*) as predicted by the Bellman Equation (4), namely

(7) $$y_{k}≔y_{k}\left(s,a,s^{\prime }\right)=r+\gamma \max_{a^{\prime }}\tilde{Q}\left(s^{\prime },a^{\prime }|\theta_{k-1}\right),$$

with the estimation of a parametrized regression model $\left(s,a\right)\mapsto \tilde{Q}\left(s,a|\theta_{k-1}\right)$. From a neurobiological perspective, experience replay can be manifested as the reoccurrence of neuron spiking sequences that have also been measured during specific prior actions or environmental states. The HC is a strong candidate for contributing to such neural reinstantiation of behavioral episodes as neuroscience experiments have repeatedly indicated in rats, mice, cats, rabbits, songbirds, and monkeys (Buhry, Azizi, & Cheng, 2011; Dave & Margoliash, 2000; Nokia, Penttonen, & Wikgren, 2010; Skaggs et al., 2007). Importantly, neural encoding of abstract representations of space and meaning may extent to several parts of the DMN (Constantinescu, O'Reilly, & Behrens, 2016; see Figure 4).

At the current step *k*, computing an optimal parameter update then corresponds to finding the model parameters *θ*_*k*_ which minimize the following mean‐squared optimization loss

(8) $$ℒ\left(\theta_{k}^{Q}\right)=E_{\left(s,a,r,s^{\prime }\right)\sim D}\left[\frac{1}{2}{\left(\tilde{Q}\left(s,a|\theta_{k}\right)-y_{k}\right)}^{2}\right],$$

where *y*_*k*_ is obtained from Equation (4). A recently proposed, practically successful alternative approach is to estimate the representation using an artificial deep neural‐network model. This approach leads to the so‐called *deep Q‐learning* (Mnih et al., 2015; Silver et al., 2016)—a family of methods which is the current state‐of‐the‐art in RL research. The set of model parameters *θ* that instantiate the nonlinear interactions between layers of the artificial neural network may find a neurobiological correspondence in the adaptive strengths of axonal connections between neurons from the different levels of the neural processing hierarchy (Mesulam, 1998; Taylor, Hobbs, Burroni, & Siegelmann, 2015).

#### 4.2.4 A note on bias in self‐training

Some bias may be introduced by self‐training due to information shortage caused by the absence of external stimulation. One way to address this issue is using importance sampling to replay especially those state‐transitions from which there is more to learn for the agent (Hessel et al., 2017; Schaul, Quan, Antonoglou, & Silver, 2015). New transitions are inserted into the replay buffer with maximum priority, thus shifting emphasis to more recent transitions. Such insertion strategy would help counterbalance the bias introduced by the information shortage incurred by absent external input. Other authors noticed (Hessel et al., 2017) that such prioritized replay reduces the data complexity and the agent shows faster increases in learning performance.

#### 4.2.5 Optimal control via stochastic gradient descent

Efficient learning of the entire set of model parameters can effectively be achieved via stochastic *gradient descent*, a universal algorithm for finding local minima based on the first derivative of the optimization objective. Stochastic here means that the gradient is estimated from batches of training samples, which here corresponds to blocks of experience from the replay memory:

(9) $$\delta =-\alpha_{k}\nabla_{\theta_{k}}ℒ\left(\theta_{k}\right)=-\alpha_{k}E_{\left(s,a,r,s^{\prime }\right)\sim D}\left[\underset{\text{prediction error}}{\underset{︸}{\left(\tilde{Q}\left(s,a|\theta_{k}\right)-y_{k}\right)}}\underset{\text{aversion}}{\underset{︸}{\nabla_{\theta_{k}}\tilde{Q}\left(s,a|\theta_{k}\right)}}\right],$$

where the positive constants *α*_1_, *α*_2_, … are learning rates. Thus, the subsequent action is taken to drive reward prediction errors to percolate from lower to higher processing layers to modulate the choice of future actions. It is known that under special conditions on the learning rates *α*_*k*_—namely, that the learning rates are neither too large nor too small, or more precisely that the sum $\sum_{k=0}^{\infty }\alpha_{k}$ diverges while $\sum_{k=0}^{\infty }\alpha_{k}^{2}$—the thus generated approximating sequence of *Q*‐value functions

$$\tilde{Q}\left(.,.|\theta_{0}\right)\to \tilde{Q}\left(.,.|\theta_{1}\right)\to \tilde{Q}\left(.,.|\theta_{2}\right)\to \ldots$$

are attracted and absorbed by the optimal *Q*‐value function Q^*^ defined implicitly by the Bellman Equation (4).

#### 4.2.6 Does the hippocampus subserve Monte Carlo sampling?

In RL, Monte Carlo simulation is a common means to update the agent's belief state based on stochastic sampling of environmental states and possible transitions (Daw & Dayan, 2014; Silver & Veness, 2010). Monte Carlo simulation provides a simple method for evaluating the value of a state. This inference procedure provides an effective mechanism both for tree search of the considered action trajectories and for belief state updates, breaking the curse of dimensionality and allowing much greater scalability than a RL agent without stochastic resampling procedures. Such methods scale as a function of available data (i.e., sample complexity) that is determined only by the underlying difficulty of the MDP, rather than the size of the state space or observation space, which can be prohibitively large.

In the human brain, the HC could contribute to synthesizing imagined sequences of world states, actions, and rewards (Aronov, Nevers, & Tank, 2017; Boyer, 2008; Chao, Nikolaus, Brandão, Huston, & de Souza Silva, 2017). These stochastic simulations of experience batches reassembled from memory would be used to update the value function, without ever looking inside the black box describing the model's dynamics. A brain‐imaging experiment in humans for instance identified hippocampal signals that specifically preceded upcoming choice performance in prospective planning in new environments (Kaplan et al., 2017). It would be a simple strategy to evaluate all legal actions and selecting the action with highest expected cumulative rewards. In MDPs, MC simulation provides an effective mechanism both for tree search and for belief‐based state updates, breaking the curse of dimensionality and allowing much greater scalability than has previously been possible (Silver et al., 2016). This is because expected consequences of action choices can be well evaluated although only a subset of the states are actually considered (Daw & Dayan, 2014).

#### 4.2.7 A note on implicit and explicit memory

While Markov processes are usually memoryless, it is mathematically feasible to incorporate a set of previous states of such model into the current state. This extension may partly account for implicit memory at the behavioral level, but may not explain the underlying neurobiological implementation or accommodate explicit memory. Implicit memory‐based processing arises in our MDP account of DMN function in several different forms: successive updates of (a) the action policy and the value function, both being products of the past, as well as (b) the deep nonlinear relationships within the hierarchical connections of biological neural networks (especially in the association cortex). The brain's adaptive synaptic connections can be viewed as a deep artificial neural‐network architecture affording an implicit form of information compression of life experience. Such memory traces are stored in the neural machinery and can be implicitly retrieved as a form of knowledge during simulation of action rather than accessed as a stored explicit representation (Pezzulo, 2011). (c) Certain neural processes in the hippocampus can be seen as some type of Monte Carlo sampling for memory recall, which can also be a basis for probabilistic simulations across time scales (Axelrod, Rees, & Bar, 2017; Schacter et al., 2007).

### 4.3 Summary and hypotheses for future studies

The DMN is today known to consistently increase in neural activity when humans engage in cognitive processes that are relatively detached from the current sensory environment. The more familiar and predictable the current environment, the more brain resources may remain for allocating DMN activity to MDP‐type processes extending beyond the present time and sensory context. This speculation receives quantitative support in that connectional links between nodes of the DMN have been reported to be more consistent and reliable than functional couplings within any other macroscopical networks (Shehzad et al., 2009). As such, random‐sampling‐related baseline evaluation of action possibilities and their consequences may be subserved by the DMN and get partly suspended when novelty in the external environment is encountered or immediate action is required (Hong, 2007; Moscovitch, Cabeza, Winocur, & Nadel, 2016). In line with this perspective, DMN engagement was shown to heighten and relate to effective behavioral responses in the practiced phase of a demanding cognitive flexibility task, as compared to acquisition phase when participants learned context‐specific rules. In major depression patients, rumination and worry may lead to a lack of novelty, not in the environment itself, but in its perception by the patient. Such examples may thus explain an abnormal activity of both DMN and the reward system. This involvement in automated decision‐making has led the authors to propose an “autopilot” role for the DMN (Vatansever, Menon, & Stamatakis, 2017), which may contribute to optimizing intervention of the organism on the world in general. Among all parts of the DMN, the RTPJ is perhaps the most evident candidate for a network‐switching relay that calibrates between processing of environment‐engaged versus internally generated information (Bzdok, Langner, Schilbach, Jakobs, et al., 2013; Downar et al., 2000; Golland et al., 2006; Kernbach et al., 2018).

Additionally, the DMN was proposed to be situated at the top of the brain network hierarchy, with the subordinate salience and dorsal attention networks in the middle and the primary sensory cortices at the bottom (Carhart‐Harris & Friston, 2010; Margulies et al., 2016). Its putative involvement in thinking about hypothetical experiences and future outcomes appears to tie in with the implicit computation of action and state cascades as a function of experienced events and collected feedback from the past. A policy matrix encapsulates the choice probabilities of possible actions on the world given a current situation (i.e., state). The DMN may subserve constant exploration of candidate action trajectories and nested estimation of their cumulative reward outcomes. Implicit computation of future choices provides a potential explanation for the evolutionary emergence and practical usefulness of mind‐wandering at day‐time and dreams during sleep in humans.

Our formal account on the DMN readily motivates several empirical predictions for future neuroscience research. Perhaps one of the first experimental venues concerns the neural correlates of the Bellman equation in the DMN. There are already relationship between the decomposition of consecutive action choices by the Bellman equation and neuroscientific insights: specific neural activity in the dorsal prefrontal cortex (BA9) was for instance linked to processing “goal‐tree sequences” in human brain‐imaging experiments (Koechlin et al., 1999; Koechlin, Corrado, Pietrini, & Grafman, 2000). Sub‐goal exploration may require multi‐task switching between cognitive processes as later parts of a solution frequently depend on respective earlier steps in a given solution path, which necessitates storage of expected intermediate outcomes. As such, “cognitive branching” operations for nested processing of behavioral strategies are likely to entail secondary reallocation of attention and working‐memory resources. Further brain‐imaging experiments corroborated the prefrontal DMN to subserve “processes related to the management and monitoring of sub‐goals while maintaining information in working memory” (Braver & Bongiolatti, 2002) and to functionally couple with the hippocampus conditioned by “deep versus shallow planning” (Kaplan et al., 2017). Moreover, neurological patients with lesions in this DMN region were reported to be impaired in aspects of realizing “multiple sub‐goal scheduling” (Burgess, Veitch, de Lacy Costello, & Shallice, 2000). Hence, the various advanced human abilities subserved by the DMN, such as planning and abstract reasoning, can be viewed to involve some form of action‐decision branching to enable higher‐order executive control.

We therefore hypothesize in humans a functional dissociation between computations pertaining to action policy versus adapting stimulus‐value associations as we expect implementation in different subsystems of the DMN. First, we expect that fMRI signals in the right temporo‐parietal junction relate to behavioral changes subsequent to adaptation in the action choice tendencies (policy matrix) involved in nonvalue‐related prediction error. Second, fMRI signals in the ventromedial prefrontal cortex should relate to behavioral changes following adaptation in value estimation (value matrix) due to reward‐related stimulus‐value association. We further expect that fMRI signals in the PMC, as a potential global information integrator, are related to shifts in overt behavior based on previous adaptations in both policy or value estimation.

Our process model of the DMN has also implications for experiments in neuroeconomy; especially for temporal discounting and continuous learning paradigms. More specifically, we hypothesize in humans a functional relationship between the DMN closely associated with the occurrence of stimulus‐independent thoughts and the reward circuitry. During an iterative neuroeconomic two‐player game, fMRI signals in the DMN could be used to predict reward‐related signals in the NAc across trials in a multi‐step learning paradigm. We expect that the more DMN activity is measured to be increased, supposedly the higher the tendency for stimulus‐independent thoughts, the more the fMRI signals in the reward circuits should be independent of the reward context in the current sensory environment. In the case of temporal discounting, we hypothesize in humans that the relevant time horizon is modulated by various factors such as age, acute stress, and time‐enduring impulsivity traits (Haushofer & Fehr, 2014; Luksys, Gerstner, & Sandi, 2009). Using such a delayed‐reward experiment, it can be quantified how the time horizon is affected at the behavioral level and then traced back to its corresponding neural representation. Such experimental investigation can be designed to examine between‐group and within‐group effects (e.g., impulsive population like chronic gamblers or drug addicts); and brought in context with the participant's age, education, IQ, and personality traits.

As another experimental prediction derived from our MDP approach to the DMN, the HC may contribute to generating perturbed action‐transition‐state‐reward samples as batches of pseudo‐experience (i.e., recalled, hypothesized, and forecasted scenarios). The small variations in these experience samplings allow searching through a larger space of model parameters and candidate experiences. Taken to its extreme, stochastic recombination of experience building blocks can further optimize the behavior of the RL agent by learning from scenarios in the environment that the agent might encounter only very rarely or never. An explanation is thus offered for experiencing seemingly familiar situations that a human has however never actually encountered (i.e., déjà vu effect). While such a situation may not have been experienced in the physical world, the DMN may have previously stochastically generated, evaluated, and adapted to such a randomly synthesized event. Generated representations arguably are “internally manipulable, and can be used for attempting actions internally, before or instead of acting in the external reality, and in diverse goal and sensory contexts, that is, even outside the context in which they were learned” (Pezzulo, 2011). In the context of scarce environmental input and feedback (e.g., mind‐wandering or sleep), mental scene construction allows pseudo‐experiencing possible future scenarios and action outcomes.

A possible interplay between memory retrieval and “mind‐searching” moreover suggests that experience replay for browsing problem solutions subserved by the DMN contributes to choice behavior in mice. Hippocampal single‐cell recordings have shown that neural patterns during experimental choice behavior are reiterated during sleep and before making analogous choices in the future. We hypothesize that, in addition to the hippocampus, there is a necessity of cortical DMN regions for “mind‐searching” candidate actions during choice behavior in humans or monkeys. It can be experimentally corroborated by causal disruption of DMN regions, such as by circumscribed brain lesion or optogenetic intervention in the inferior parietal and prefrontal cortices. From the perspective of a RL agent, prediction in the DMN reduces to generalization of policy and value computations from sampled experiences to successful action choices and reward predictions in future states. As such, plasticity in the DMN arises naturally. If an agent behaving optimally in a certain environment moves to a new, never experienced environment, reward prediction errors will largely increase. This feedback will lead to adaptation of policy considerations and value estimations until the intelligent system converges to a new steady state of optimal action decisions in a volatile world.

A last experimental prediction for future studies concerns how synaptic epigenesis may shape the policy matrix. Indeed, we did not address the additional layer of learning which concerns the addition of new entries in the state and action spaces. Extension of the action repertoire could be biologically realized by synaptic epigenesis (Gisiger, Kerszberg, & Changeux, 2005). The tuning of synaptic weights through learning can stabilize additional patterns of activity by creating new attractors in the neural dynamics landscape (Takeuchi, Duszkiewicz, & Morris, 2014). Those attractors can then constrain both the number of factors taken into account by decision processes and the possible behaviors of the agent (Wang, 2008). To examine this potential higher‐level mechanism, we propose to probe how synaptic epigenesis is related to neural correlates underlying policy matrix updates: in humans the changes of functional connectivity between DMN regions can be investigated following a temporal discounting experiment and in monkeys or rodents anterograde tracing can be used to study how homolog regions of the DMN present increased synaptic changes compare to other parts of the brain.

## 5 RELATION TO EXISTING ACCOUNTS

### 5.1 Predictive coding

Predictive coding mechanisms (Clark, 2013; Friston, 2008) are a frequently evoked idea in the context of default mode function (Bar, Aminoff, Mason, & Fenske, 2007). Cortical responses are explained as emerging from continuous functional interaction between higher and lower levels of the neural processing hierarchy. Feed‐forward sensory processing is constantly calibrated by top‐down modulation from more multi‐sensory and associative brain regions further away from primary sensory cortical regions. The dynamic interplay between cortical processing levels may enable learning about aspects of the world by reconciling gaps between fresh sensory input and predictions computed based on stored prior information. At each stage of neural processing, an internally generated expectation of aspects of environmental sensations is directly compared against the actual environmental input. A prediction error at one of the processing levels induces plasticity changes of neuronal projections to allow for gradually improved future prediction of the environment. In this way, the predictive coding hypothesis offers explanations for the constructive, nondeterministic nature of sensory perception (Buzsáki, 2006; Friston, 2010) and the intimate relation of motor movement to sensory expectations (Kording & Wolpert, 2004; Wolpert, Ghahramani, & Jordan, 1995). Contextual integration of sensorimotor perception‐action cycles may be maintained by top‐down modulation using internally generated information about the environment.

**FIGURE 5:**
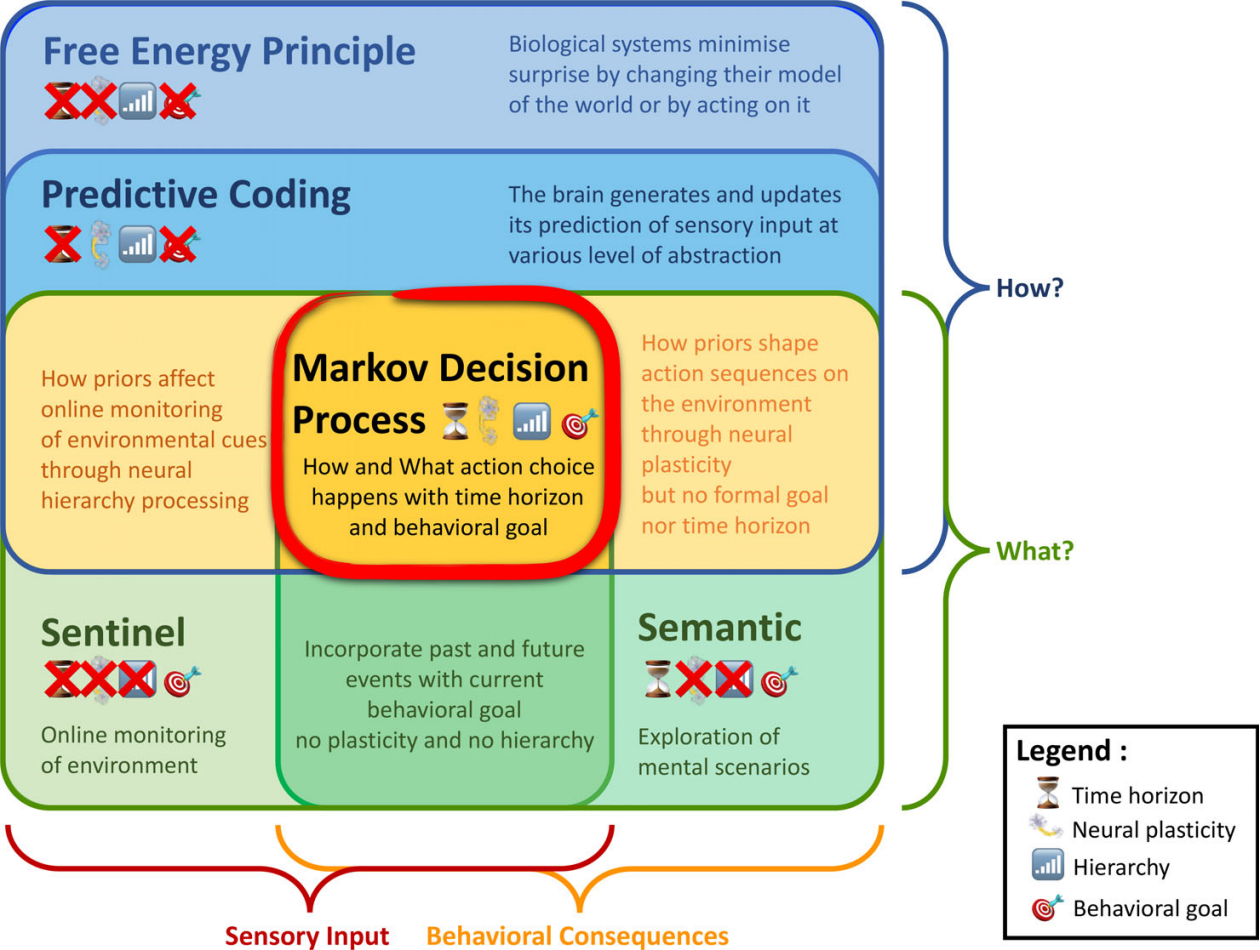
Situating Markov decision processes among other accounts of default mode function. The Venn diagram summarizes the relationship between four previously proposed explanations for the functional role of the DMN and our present account. Viewing empirical findings in the DMN from the MDP viewpoint incorporates important aspects of the free energy principle, predictive coding, sentinal hypothesis, and semantic hypothesis. The MDP account may reconcile several strengths of these functional accounts in a process model that simultaneously acknowledges environmental input and behavioral choices as well as the computational and algorithmic properties (How? and What?) underlying higher‐order control of the organism

In short, predictive coding processes conceptualize updates of the internal representation of the environment to best accommodate and prepare the organism for processing the constant influx of sensory stimuli and performing action on the environment (Figure 5). There are hence a number of common properties between the predictive coding account and the proposed formal account of DMN function based on MDPs. Importantly, a generative model of how perceived sensory cues arise in the world would be incorporated into the current neuronal wiring. Further, both functional accounts are supported by neuroscientific evidence that suggest the human brain to be a “statistical organ” (Friston, Stephan, Montague, & Dolan, 2014) with the biological purpose to generalize from the past to new experiences. Neuroanatomically, axonal back projections indeed outnumber by far the axonal connections mediating feedforward input processing in the monkey brain and probably also in humans (Salin & Bullier, 1995). These many and diverse top‐down modulations from higher onto downstream cortical areas can inject prior knowledge at every stage of processing environmental information. Moreover, both accounts provide a parsimonious explanation for why the human brain's processing load devoted to incoming information decreases when the environment becomes predictable. This is because the internal generative model only requires updates after discrepancies have occurred between environmental reality and its internally reinstantiated representation. Increased computation resources are however allocated when unknown stimuli or unexpected events are encountered by the organism. The predictive coding and MDP accounts hence naturally evoke a mechanism of brain plasticity in that neuronal wiring gets increasingly adapted when faced by unanticipated environmental challenges.

While sensory experience is a constructive process from both views, the predictive coding account frames sensory perception of the external world as a generative experience due to the modulatory top‐down influence at various stages of sensory input processing. This generative top‐down design is replaced in our MDP view of the DMN by a sequential decision‐making framework. Further, the hierarchical processing aspect from predictive coding is re‐expressed in our account in the form of nested prediction of probable upcoming actions, states, and outcomes. While both accounts capture the consequences of action, the predictive coding account is typically explained without explicit parameterization of the agent's time horizon and has a tendency to be presented as emphasizing prediction about the immediate future. In the present account, the horizon of that look into the future is made explicit in the *γ* parameter of the Bellman equation.

Finally, the process of adapting the neuronal connections for improved top‐down modulation takes the concrete form of stochastic gradient computation and back‐propagation in our MDP implementation. It is however important to note that the neurobiological plausibility of the back‐propagation procedure is controversial (Goodfellow, Bengio, & Courville, 2016).

In sum, recasting DMN function in terms of MDPs therefore naturally incorporates the majority of aspects from the prediction coding hypothesis. The present MDP account of DMN function may therefore serve as a concrete implementation of many predictive coding ideas. MDPs have the advantage of exposing an explicit mechanisms for modulating the horizon of future considerations and for how the internal representation of the world is updated, as well as why certain predictions may be more relevant to the agent than others.

### 5.2 The semantic account

This frequently embraced cognitive account to explain DMN function revolves around forming logical associations and abstract analogies between experiences and conceptual knowledge derived from past behavior (Bar, 2007; Binder et al., 1999; Constantinescu, O'Reilly, & Behrens, 2016b). Analogies might naturally tie incoming new sensory stimuli to explicit world knowledge (i.e., semantics, Figure 5; Bar, 2009). The encoding of complex environmental features could thus be facilitated by association to known similar states. Going beyond isolated meaning and concepts extracted from the world, semantic building blocks may need to get recombined to enable mental imagery to (fore)see never‐experienced scenarios. As such, semantic knowledge would be an important ingredient for optimizing behavior by constantly simulating possible future scenarios (Binder & Desai, 2011; Boyer, 2008). Such cognitive processes can afford the internal construction and elaboration of necessary information that is not presented in the immediate sensory environment by recombining building blocks of concept knowledge and episodic memories (Hassabis & Maguire, 2009). Indeed, in aging humans, remembering the past and imagining the future equally decreased in the level of detail and were associated with concurrent deficits in forming and integrating relationships between items (Addis, Wong, & Schacter, 2008; Spreng & Levine, 2006).

Further, episodic memory, language, problem solving, planning, estimating others' thoughts, and spatial navigation represent neural processes that are likely to build on abstract world knowledge and logical associations for integrating the constituent elements in rich and coherent mental scenes (Schacter et al., 2007). “[Foresight] and simulations are not only automatically elicited by external events but can be endogenously generated when needed. […] The mechanism of access via simulation could be a widespread method for accessing and producing knowledge, and represents a valid alternative to the traditional idea of storage and retrieval” (Pezzulo, 2011). Such mental scene‐construction processes could contribute to interpreting the present and foreseeing the future. Further, mental scene imagery has been proposed to imply a distinction between engagement in the sensory environment and internally generated mind‐wandering (Buckner & Carroll, 2007). These investigators stated that “A computational model […] will probably require a form of regulation by which perception of the current world is suppressed while simulation of possible alternatives are constructed, followed by a return to perception of the present.”

In comparison, both the semantic hypothesis and the present formal account based on MDPs expose mechanisms of how action considerations could be explored. In both accounts, there is also little reason to assume that contemplating alternative realities of various levels of complexity, abstraction, time scale, and purpose rely on mechanisms that are necessarily qualitatively different. This interpretation concurs with DMN activity increases across time, space, and content domains demonstrated in many brain‐imaging studies (Binder et al., 2009; Bzdok et al., 2012; Laird et al., 2009; Spreng et al., 2009). Further, the semantic hypothesis and MDP account offer explanations why HC damage does not only impair recalling past events, but also imagining hypothetical and future scenarios (Hassabis et al., 2007). While both semantic hypothesis and our formal account propose memory‐enabled, internally generated information for probabilistic representation of action outcomes, MDPs render explicit the grounds on which an action is eventually chosen, namely, the estimated cumulative reward. In contrast to many versions of the semantic hypothesis, the MDPs naturally integrate the egocentric view (more related to current action, state, and reward) and the world view (more related to past and future actions, states, and rewards) on the world in a same optimization problem. Finally, the semantic account of DMN function does not provide suffcient explanation of *how* explicit world knowledge and logical analogies thereof lead to foresight of future actions and states. The semantic hypothesis does also not fully explain why memory recall for scene construction in humans is typically fragmentary and noisy instead of accurate and reliable. In contrast to existing accounts on semantics and mental scene construction, the random and creative aspects of DMN function are explained in MDPs by the advantages of stochastic optimization. Our MDP account provides an algorithmic explanation in that stochasticity of the parameter space exploration by Monte Carlo approximation achieves better fine‐tuning of the action policies and inference of expected reward outcomes. That is, the purposeful stochasticity of policy and value updates in MDPs provides a candidate explanation for why humans may have evolved imperfect noisy memories as the more advantageous adaptation. In sum, mental scene construction according to the semantic account is lacking explicit time and incentive structure, both of which are integral parts of the MDP interpretation of DMN function.

### 5.3 The sentinel account

Regions of the DMN have been proposed to process the experienced or expected relevance of environment cues (Montague, King‐Casas, & Cohen, 2006). Processing self‐relevant information was perhaps the first functional account that was proposed for the DMN (Gusnard, Akbudak, Shulman, & Raichle, 2001; Raichle et al., 2001). Since then, many investigators have speculated that neural activity in the DMN may reflect the brain's continuous tracking of relevance in the environment, such as spotting predators, as an advantageous evolutionary adaptation (Buckner et al., 2008; Hahn, Ross, & Stein, 2007). According to this cognitive account, the human brain's baseline maintains a “radar” function to detect subjectively relevant cues and unexpected events in the environment (Figure 5). Propositions of a sentinel function to underlie DMN activity have however seldom detailed the mechanisms of how attention and memory resources are exactly reallocated when encountering a self‐relevant environmental stimulus. Instead, in the present MDP account, promising action trajectories are recursively explored by the human DMN. Conversely, certain branches of candidate action trajectories are detected to be less worthy to be explored. This mechanism, expressed by the Bellman equation, directly implies stratified allocation of attention resources and working memory load over relevant cues and events in the environment.

Further, our account provides a parsimonious explanation for the consistently observed DMN implication in certain goal‐directed experimental tasks and in task‐unconstrained mind‐wandering (Bzdok et al., 2016; Smith et al., 2009). Both environment‐detached and environment‐engaged cognitive processes may entail DMN recruitment if real or imagined experience is processed, manipulated, and used in service of organism control. During active engagement in tasks, the policy and value estimates may be updated to optimize especially short‐term action. At passive rest, these parameter updates may improve especially mid‐and long‐term action. This horizon of the agent is expressed in the *γ* parameter in the MDP account. We thus provide answers for the currently unsettled question why the involvement of the same neurobiological brain circuit (i.e., DMN) has been documented for specific task performances and baseline “house‐keeping” functions.

In particular, environmental cues that are especially important for humans are frequently of social nature. This may not be surprising given that the complexity of the social systems is likely to be a human‐defining property (Dunbar & Shultz, 2007; Kiesow et al., 2020; Tomasello, 2009). According to the “social brain hypothesis,” the human brain has especially been shaped for forming and maintaining increasingly complex social systems, which allows solving ecological problems by means of social relationships (Whiten & Byrne, 1988). In fact, social topics probably amount to roughly two‐thirds of human everyday communication (Dunbar, Marriott, & Duncan, 1997). Mind‐wandering at daytime and dreams during sleep are also rich in stories about people and the complex interactions between them. In line with this, DMN activity was advocated to be specialized in continuous processing of social information as a physiological baseline of human brain function (Schilbach, Eickhoff, Rotarska‐Jagiela, Fink, & Vogeley, 2008). This view was later challenged by observing analogues or protoforms of the DMN in monkeys (Mantini et al., 2011), cats (Popa, Popescu, & Paré, 2009), and rats (Lu et al., 2012), three species with social capacities that can be expected to be less advanced than in humans (Mars et al., 2012).

Moreover, the principal connectivity gradient in the cortex appears to be greatly expanded in humans compared to monkeys, suggesting a phylogenetically conserved axis of cortical expansion with the DMN emerging at the extreme end in humans (Margulies et al., 2016). Computational models of dyadic whole‐brain dynamics demonstrated how the human connectivity topology, on top of facilitating processing at the intraindividual level, can explain our propensity to coordinate through sensorimotor loops with others at the inter‐individual level (Dumas, Chavez, Nadel, & Martinerie, 2012). The DMN is moreover largely overlapping with neural networks associated with higher‐level social processes (Alcalá‐López et al., 2018; Schilbach et al., 2012). For instance, the vmPFC, PMC, and RTPJ together may play a key role in bridging the gap between self and other by integrating low‐level embodied processes within higher level inference‐based mentalizing (Alcalá‐López et al., 2017; Lombardo et al., 2009).

Rather than functional specificity for processing social information in particular, the present MDP account can parsimoniously incorporate the dominance of social content in human mental activity as high value function estimates given the general relevance of information about humans (Baker, Saxe, & Tenenbaum, 2009; Bzdok et al., 2011; Kampe, Frith, Dolan, & Frith, 2001; Krienen, Tu, & Buckner, 2010). The DMN may thus modulate reward processing in the human agent in a way that prioritizes appraisal of and action toward social contexts, without excluding relevance of environmental cues of the physical world. In sum, our account on the DMN directly implies its previously proposed “sentinel” function of monitoring the environment for self‐relevant information in general and inherently accommodates the importance of social environmental cues as a special case.

### 5.4 A note on the free‐energy principle and active inference

According the *free‐energy principle* (FEP) and theories of *active inference* (Dayan, Hinton, Neal, & Zemel, 1995; Friston, 2010; Friston, Daunizeau, & Kiebel, 2009), the brain corresponds to a biomechanical reasoning engine. Much of neural computation is dedicated to minimizing the long‐term average of surprise: the log‐likelihood of the observed sensory input—more precisely, an upper bound thereof—relative to the expectations about the external world derived from internal representations. The brain would continuously generate hypothetical explanations of the world and predict its sensory input *x* (analogs to the state‐action (*s*, *a*) pair in an MDP framework).

However, surprise is challenging to optimize numerically because we need to solve the intractable problems of summing over all hidden causes **z** of the sensations. Instead, FEP therefore minimizes an upper‐bound on surprise given by

(10) $$\begin{array}{l} \text{generative surprise}≔-\log\left(p_{G}\left(x\right)\right)=F_{G}\left(x\right)\\ \;=\underset{\text{accuracy}}{\underset{︸}{F_{G}^{R}\left(x\right)}}-\underset{\text{complexity}}{\underset{︸}{\mathrm{KL}\left(p_{R}\left(z|x\right)\|p_{G}\left(z|x\right)\right)}}\\ \;\leq F_{G}^{R}\left(x\right),\text{with equality if}\;p_{R}\left(z|x\right)=p_{G}\left(z|x\right)\;\text{for}\;\mathrm{all}\;z. \end{array}$$

where

(11) $$F_{G}^{R}\left(x\right)≔{〈-\log\left(p_{G}\left(z,x\right)\right)〉}_{p_{R}\left(z|x\right)}-ℋ\left(p_{R}\left(z|x\right)\right)$$

is the *free energy*. Here, the angular brackets denote the *expectation* of the joint negative log‐likelihood −log(*p*_*G*_(**z**, **x**)) w.r.t the recognition density *p*_*R*_(**z**|**x**), ℋ is the *entropy* function defined by $ℋ\left(p\right)≔-\sum_{z}p\left(z\right)\log(p\left(z\right)$, while KL(.∥.) is the usual *Kullback–Leibler (KL) divergence* (also known as *relative entropy*) defined by $\mathrm{KL}\left(p\|q\right)≔\sum_{z}p\left(z\right)\log\left(p\left(z\right)/q\left(z\right)\right)\geq 0$, which is a measure of difference between two probability distributions. In this framework, the goal of the agent is to iteratively refine the generative model *p*_*G*_ and the recognition model *p*_*R*_ so as to minimize the free energy $F_{G}^{R}\left(x\right)$ over sensory input **x**.

Importantly, $F_{G}^{R}\left(x\right)$ gets low in the following cases:

- *p*_*R*_(**z**|**x**) puts a lot of mass on configurations (**z**, **x**) which are *p*_*G*_‐likely
- *p*_*R*_(**z**|**x**) is as uniform as possible (i.e., have high entropy), so as not to concentrate all its mass on a small subset of possible causes for the sensation **x**

Despite its popularity, criticism against the FEP has been voiced repeatedly, which we allude to in the following. The main algorithm for minimizing free energy $F_{G}^{R}\left(x\right)$ is the *wake–sleep algorithm* (Dayan et al., 1995). As these authors noted, a crucial drawback of the wake–sleep algorithm (and therefore of theories like the FEP [Friston, 2010]) is that it involves a pair of forward (generation) and backward (recognition) models *p*_*G*_ and *p*_*R*_ that together does not correspond to optimization of a bound of the marginal likelihood because KL divergence is not symmetric in its arguments.

These considerations may render the brain less likely to implement a variant of the wake–sleep algorithm. More recently, *variational auto‐encoders* (Kingma & Welling, 2013) emerged that may provide an efficient alternative to the wake–sleep algorithm. Such compression‐and‐reconstruction models overcome a number of the technical limits of the wake–sleep algorithm by using a reparametrization maneuver, which makes it possible to do differential calculus on random sampling procedures without exploding variance. As a result, unlike the wake–sleep algorithm for minimizing free energy, variational auto‐encoders can be efficiently trained via back‐propagation of prediction errors.

The difference between the FEP and the MDP account may be further clarified by a thought experiment. Since theories based on the FEP (Friston, 2010; Friston et al., 2009) conceptualize ongoing behavior in an organism to be geared toward the surprise‐minimizing goal. Hence, an organism entering a dark room would remain trapped in this location because its sensory inputs are perfectly predictable given the environmental state (Friston, Thornton, & Clark, 2012). However, such a behavior is seldom observed in humans in the real world. In a dark room, the intelligent agents would search for light sources to explore the surroundings or aim to exit the room.

One may object that, for the FEP agent, a dark room would paradoxically correspond to a state of particularly high relevance. Driven by the surprise‐minimization objective, the FEP agent would eventually not bootstrap itself out of such saddle points to explore more interesting parts of the environment. In contrast, an organism operating under our RL‐based theory would inevitably identify the sensory‐stimulus‐deprived room as a local minimum. Indeed, hippocampal experience replay (see Section 4.2.3) could serve to sample memories or fantasies of alternative situations with reward structure. Such artificially generated *internal* sensory input, potentially subserved by the DMN, could then entice the organism to explore the room, for instance by looking for and using the light switch or finding the room exit.

We finally note that FEP and active inference can be reframed in terms of our RL framework. This is possible by recasting the Q‐value function (i.e., expected long‐term reward) potentially maximized by the DMN to correspond to negative surprise, that is, the log‐likehood of current sensory priors the agent has about the world. More explicitly, this formulation corresponds to using free‐energy as a *Q*‐value approximator for the MDP in the following way:

$$-Q\;\approx \;\underset{\text{negative free energy}}{\underset{︸}{F_{G}^{R}\left(x\right)}}\;\approx \;\underset{\mathrm{FEP}\;\text{generative surprise}}{\underset{︸}{-\log\left(p_{G}\right)}}.$$

Such a surprise‐guided RL scheme has previously been advocated under the equivalent framework of energy‐based RL.

(Elfwing, Uchibe, & Doya, 2016; Sallans & Hinton, 2004) and information compression (Mohamed & Rezende, 2015; Schmidhuber, 2010). More broadly, minimization of surprise quantities alone may be insufficient to explain the diversity of behaviors that humans and other intelligent animals are able to perform.

## 6 CONCLUSION

Which brain function could be important enough for the existence and survival of the human species to justify constantly high energy costs? While previous experiments on the DMN frequently set out to investigate *what* its subserved function may be, we have proposed a way of reasoning *how* this major brain network may do what it is doing. MDPs motivate an attractive formal account of how the association cortex, expanded so much in the human brain, can be thought to implement multi‐sensory representation and high‐level decision‐making to optimize the organism's behavioral strategies. This idealized process model accommodates a number of previous observations from neuroscience studies on the DMN by simple but nontrivial mechanisms. Viewed as a Markovian sequential decision process, human behavior unfolds by inferring expected reward outcomes from hypothetical action cascades and extrapolation from past experience to upcoming events for guiding behavior in the present. MDPs also provide a formalism how opportunity in the environment can be deconstructed, evaluated, and exploited when an agent is confronted with challenging interdependent decisions. This abstract process interpretation may well be compatible with the DMN's poorly understood functional involvement across autobiographical memory recall, problem solving, abstract reasoning, social cognition, as well as delay discounting and self‐prospection into the future. For instance, improvement of the internal world representation by injecting stochasticity into the recall of past actions and inference of action outcomes may explain why highly accurate memories have been disfavored in human evolution and why human creativity is adaptive.

A major hurdle in guessing DMN function from cognitive brain‐imaging studies has been its similar neural engagement in different time scales: thinking about the past (e.g., autobiographical memory retrieval), imagining hypothetical presents (e.g., daytime mind‐wandering), and anticipating scenarios yet to come (e.g., delay discounting). The MDP account of DMN activity offers a natural integration of a‐priori diverging classes of cognitive processes into a common framework. It is an important advantage of the proposed artificial intelligence perspective on DMN biology that it is practically computable and readily motivates neuroscientific hypotheses that can be put to the test in future research. We encourage neuroscience experiments on the DMN to operationalize the set of action, value, and state variables that govern the behavior of intelligent RL agents. At the least, we propose an alternative vocabulary to describe, contextualize, and interpret experimental findings in neuroscience studies on higher‐level cognition. Ultimately, neural processes in the DMN may realize a brain‐wide information integration ranging from real experience over purposeful dreams to predicted futures to continuously refine the organism's intervention on the world.

## Data Availability

The code for reproduction and visualization: www.github.com/banilo/darkcontrol_2018.
